# Correction: Lipid-based nanoparticles for psoriasis treatment: a review on conventional treatments, recent works, and future prospects

**DOI:** 10.1039/d2ra90076a

**Published:** 2022-08-02

**Authors:** Ummu Umaimah Mohd Nordin, Noraini Ahmad, Norazlinaliza Salim, Nor Saadah Mohd Yusof

**Affiliations:** Department of Chemistry, Faculty of Science, University of Malaya 50603 Kuala Lumpur Malaysia ainie@um.edu.my +603-79674193 +603-79674008; Integrated Chemical Biophysics Research, Faculty of Science, Universiti Putra Malaysia 43400 UPM Serdang Selangor Malaysia

## Abstract

Correction for ‘Lipid-based nanoparticles for psoriasis treatment: a review on conventional treatments, recent works, and future prospects’ by Ummu Umaimah Mohd Nordin *et al.*, *RSC Adv.*, 2021, **11**, 29080–29101, https://doi.org/10.1039/D1RA06087B.

The authors regret that an incorrect grant number was shown in the Acknowledgements section of the published article. The corrected section should read:

The authors gratefully acknowledge the Ministry of Higher Education Malaysia (MOHE) for the research project financial support through the Fundamental Research Grant Scheme (FRGS/1/2019/STG01/UM/02/7) and the University of Malaya.

 

The Royal Society of Chemistry apologises for these errors and any consequent inconvenience to authors and readers.

## Supplementary Material

